# Gender-Based Clinical Differences in Hymenoptera Venom Poisoning: A Retrospective Study From Taiwan (April 2021 to March 2023)

**DOI:** 10.1155/emmi/8893175

**Published:** 2025-06-02

**Authors:** Ching-Hsiang Yu, Sheng-Teck Tan, Hsiu-Wu Yang, Yen-Chun Lai, Yu-Jang Su

**Affiliations:** ^1^Department of Emergency Medicine, Mackay Memorial Hospital, Zhongshan District, Taipei 10449, Taiwan; ^2^Toxicology Division, Emergency Department, Mackay Memorial Hospital, Zhongshan District, Taipei 10449, Taiwan; ^3^Department of Anesthesiology, Taipei Medical University Hospital, Xinyi District, Taipei 110, Taiwan; ^4^Department of Nursing, Yuanpei University of Medical Technology, Xiangshan District, Hsinchu 300, Taiwan; ^5^Department of Medicine, Mackay Medical College, Sanzhi District, New Taipei 252005, Taiwan; ^6^Department of Nursing, Mackay Junior College of Medicine Nursing and Management, Sanzhi District, New Taipei 252005, Taiwan

**Keywords:** Hymenoptera, insect bites and stings, sex characteristics, Taiwan, venom

## Abstract

**Background:** Hymenoptera stings are a common cause of emergency visits.

**Objective:** This study aims to assess potential gender disparities in clinical presentation and outcomes of Hymenoptera stings.

**Methods:** Medical records were collected from a single medical center in Northern Taiwan, covering the period from April 1, 2021, to March 31, 2023. A total of 87 patients with confirmed Hymenoptera sting incidents were identified. Data on gender, sting location, clinical presentation, diagnostic evaluation, complications, treatment, and clinical outcomes were analyzed.

**Results:** Among the 87 patients, 47.1% were male and 52.9% were female, showing a nearly balanced distribution of cases. Females experienced a higher rate of stings during holidays. Males, however, were more likely to suffer from severe systemic reactions and had a higher average number of stings compared to females (1.3 vs. 1.0, *p*=0.049).

**Conclusion:** Males are at higher risk for multiple stings and severe systemic reactions from Hymenoptera stings. Additionally, females tend to experience more stings during spring and autumn compared to males.

## 1. Introduction

Hymenoptera stings, mainly from bees, wasps, and hornets, are a common cause of envenomation worldwide, with prevalence rates ranging from 56.6% to 94.5% in the general population, according to various studies [1]. Bees, wasps, and hornets are all members of the order Hymenoptera, but they differ in appearance, behavior, and venom potency. Bees are hairy and generally nonaggressive, stinging only once with a barbed stinger. Wasps have smooth bodies, can sting multiple times, and are more aggressive. Hornets, a type of large wasp, are the most aggressive and deliver more potent venom in greater quantities, often causing more severe systemic reactions (SRs).

While most stings lead to mild local reactions, systemic toxicity occurs in 0.4%–8% of cases, presenting as symptoms such as flushing, urticaria, angioedema, dizziness, dyspnea, nausea, and, in severe cases, life-threatening anaphylaxis [2, 3]. European epidemiological studies report that self-reported severe SRs in adults range from 0.3% to 7.5%, while in the United States, the prevalence of severe SRs is estimated between 0.5% and 3.3%. This results in approximately 220,000 emergency department visits and nearly 60 deaths annually due to Hymenoptera stings [4–6]. Additionally, studies suggest that warmer southern regions experience higher sting frequencies and sensitization rates compared to cooler climates [7]. As a result, tropical and subtropical Asian countries, such as China, India, Sri Lanka, Vietnam, Thailand, Malaysia, and Nepal, report an increased incidence of life-threatening toxic reactions from Hymenoptera stings [8, 9].

These studies highlight the global significance of Hymenoptera stings as a serious public health concern. In response, numerous researchers have worked to identify risk factors for severe SRs. Established risk factors include a history of severe reactions, mast cell disorders, cardiovascular disease, honeybee allergies, advanced age, physical exertion, alcohol consumption, stress, and the use of beta-blockers, all of which are known to increase the severity of anaphylaxis [10]. Recent epidemiological data from the Middle East also highlight the regional burden of Hymenoptera stings. In a large-scale Iranian study conducted by Afzali et al., 1430 cases of Hymenoptera stings were analyzed over a 5-year period in Southwestern Iran, a region with a hot and arid climate [11]. The study revealed that wasp stings were the most frequent (67.3%), followed by bee stings (25.9%) and hornet stings (6.8%). Notably, SRs occurred in 11.2% of patients, a rate higher than that reported in many Western studies. The most common systemic manifestations included dizziness, generalized urticaria, dyspnea, and hypotension. The study also noted that rural residents and males had a higher risk of severe reactions, consistent with occupational exposure patterns in agricultural settings. This study emphasizes the need to consider local environmental and sociodemographic factors when assessing sting-related risk and tailoring public health responses.

However, in clinical practice, particularly in overcrowded emergency departments, it can be challenging for healthcare professionals to gather detailed patient histories. Given these limitations, our focus is on easily accessible information, such as gender, to explore potential differences in clinical presentation and outcomes.

## 2. Materials and Methods

**Figure 1:**
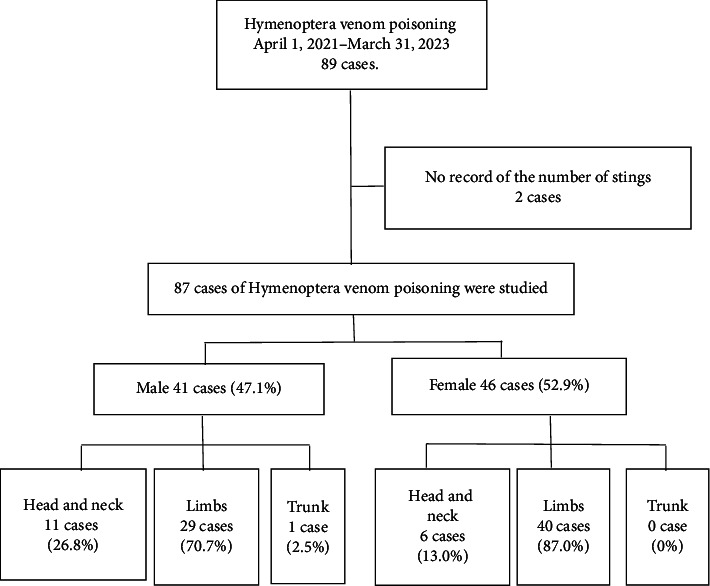
The 2-year cases of Hymenoptera venom poisoning are divided into gender and sting sites of these 87 cases.

This study employs a retrospective design, analyzing medical records from a single medical center in Northern Taiwan between April 1, 2021, and March 31, 2023 (Figure 1). A total of 89 patients with documented Hymenoptera insect stings were identified by screening the hospital information system using the International Classification of Diseases (ICD)-10 code T6344xx. Two patients were excluded due to missing records of the number of stings.

The remaining 87 patients were categorized by gender, and within each gender group, further subdivided based on the sting location: head and neck (H), limbs (L), and trunk (T). Detailed data, including demographics (gender, age, medical history, and length of stay), sting characteristics (location and number), clinical presentations (such as angioedema, bronchospasm, and anaphylactic shock), diagnostic evaluations (vital signs, biochemistry, and complete blood count), complications, treatments administered (e.g., adrenaline 0.3 mg, diphenhydramine 30 mg, and chlorpheniramine 5 mg), and clinical outcomes (duration of emergency room observation, admission to general ward, and intensive care unit (ICU)), were meticulously collected for analysis.

Ethical approval for this study was granted by the Institutional Review Board of MacKay Memorial Hospital (approval reference no. 24MMHIS003e). Since this is a retrospective study, we received a waiver of the need for informed consent from participants.

Statistical analyses were performed using commercial software. Student's *t*-tests and analysis of variance (ANOVA) were applied to determine significance. Categorical variables were compared using chi-square or Fisher's exact test, where appropriate, and results were expressed as percentages. A two-tailed *p* value of < 0.05 was considered statistically significant. Data analysis was conducted using SPSS Version 25.0 (IBM Corp., Armonk, NY, USA).

## 3. Results

**Figure 2:**
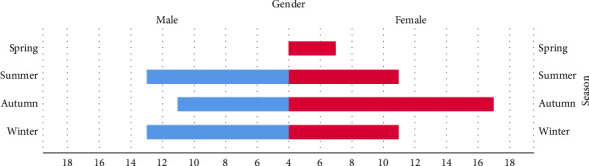
We found that female patients have a higher rate of Hymenoptera stings in spring and autumn than males, *p*=0.011 and 0.017, respectively.

Among the 87 patients included in the study, 41 were male (47.1%) and 46 were female (52.9%). There was no significant age difference between the genders (male: 44.8 ± 18.5 years, female: 48.0 ± 16.0 years, *p*=0.282). However, a significantly higher rate of bee stings occurred in females during spring and autumn compared to males, and females experienced more stings on holidays compared to weekdays, whereas males did not (47.8% vs. 26.8%, *p*=0.008) (Figure 2). Despite this, males experienced a greater number of stings than females (male: 1.3 ± 0.7, female: 1.0 ± 0.2, *p*=0.049).

**Figure 3:**
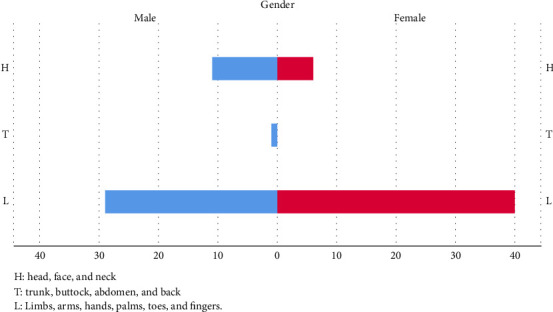
For the sting site analyses, there is no statistical difference between genders. Limbs (79.3%) are still the most commonly attacked part in both sexes.

Regarding sting locations, both males and females were most commonly stung on their L (males: 70.7%, females: 87%) (Figure 3). The second most common location was the H, with 26.8% of males and 13% of females reporting stings in this area. Only one male (2.5%) experienced stings on the T, while no females reported stings in this region. However, there was no statistically significant gender difference in the location of stings.

In terms of treatment, significant gender differences were found in the AB group (adrenaline 0.3 mg + diphenhydramine 30 mg) and the BC group (Benadryl (diphenhydramine) 30 mg + chlorpheniramine 5 mg), primarily because no females were treated in these groups. There were no gender differences observed in other treatment groups (Benadryl (diphenhydramine) 30 mg, *p*=0.328; chlorpheniramine 5 mg, *p*=0.490; no treatment, *p*=0.561) (Table 1).

**Figure 4:**
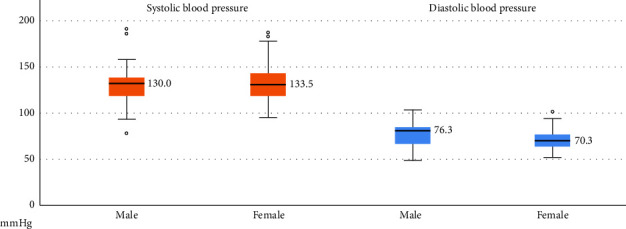
Relative lower diastolic blood pressure noted in female Hymenoptera sting cases.

Regarding vital signs and length of stay, females had significantly lower diastolic blood pressure (DBP) than males (male: 76.3 ± 12.1 mmHg, female: 70.3 ± 10.4 mmHg, *p*=0.0014) (Figure 4). However, no significant differences were observed in other vital sign parameters, including systolic blood pressure (SBP), heart rate, and body temperature. The length of stay, considered an indicator of severity, also did not differ significantly between males and females.

## 4. Discussion

This retrospective study investigated gender differences in clinical features and outcomes of patients presenting with Hymenoptera venom poisoning (HVP) at a tertiary medical center in Northern Taiwan between April 2021 and March 2023. A total of 87 patients were included. The study found that men experienced a significantly higher number of stings and elevated levels of creatine kinase (CK), alanine aminotransferase (ALT), creatinine, and potassium compared to women. SRs and complications such as rhabdomyolysis, acute kidney injury (AKI), and acute myocardial injury were also more common in men. Despite these differences in clinical severity, there was no statistically significant difference in mortality between genders. The findings highlight the importance of recognizing gender-specific clinical presentations in HVP, which may inform more tailored approaches to early diagnosis and management.

### 4.1. Gender-Based Disparities in Hymenoptera Sting Incidents in Northern Taiwan

The examination of gender differences in Hymenoptera stings has revealed intriguing insights, underscoring the complex interplay of biological, environmental, and sociocultural factors. Our findings show a nearly equal distribution of stings, with 47.1% of cases involving males and 52.9% involving females. This contrasts with studies from Korea, Brazil, and Switzerland, where a significantly higher proportion of males were affected. This discrepancy may be explained by the greater involvement of males in the beekeeping industry in those regions, increasing their risk of bee stings [11–13]. However, in Taiwan, the beekeeping industry is primarily concentrated in the central and southern regions, including Taichung, Kaohsiung, Nantou, and Chiayi, areas known for longan and lychee cultivation [14, 15]. Since our hospital is located in Northern Taiwan, the occurrence of Hymenoptera stings in this study is not linked to beekeeping activities.

### 4.2. Females Have a Higher Rate of Hymenoptera Stings on Holidays

A Swiss study found that Hymenoptera stings are more likely to occur on long, dry, warm days with high air pressure [12]. Similarly, research conducted in Brazil revealed a positive correlation between the number of bee sting reports and the average minimum temperature of the month [13]. These findings align with studies from Korea and Taiwan, which also report a higher prevalence of Hymenoptera stings during the summer and autumn months [14, 16]. A report from Northern Iran describing most Hymenoptera sting incidents occurred during the summer season, with the highest frequency around noon. This suggests a seasonal and diurnal pattern likely influenced by increased insect activity and human outdoor exposure during warm midday hours [17].

However, no study has systematically categorized patients by gender to compare seasonal distribution differences. According to the 2022 Survey of Travel by Taiwan Citizens, 65.8% of respondents planned trips during holidays, with a nearly equal gender distribution (males: 49.6%, females: 50.4%). Additionally, 64.0% engaged in outdoor activities such as sightseeing, which may increase exposure to Hymenoptera [18]. Our study found that a significant percentage of stings in females occurred on holidays (47.8% vs. 26.8%, *p*=0.008), potentially due to the use of perfumes and bright, colorful clothing. Bees and certain wasps, which serve as nectar feeders and pollinators, are drawn to vibrant, fragrant flowers [19]. As a result, wearing fragrances and brightly colored clothing, especially in shades of yellow, pink, and red, may increase the risk of Hymenoptera stings [20].

### 4.3. Gender Influences on Seasonal and Environmental Risk Factors for Hymenoptera Stings

In our study, one male received a 0.3 mg adrenalin injection. In clinical practice, adrenalin is administered to Hymenoptera sting patients exclusively in cases of anaphylactic shock [21]. Our results suggest that males may exhibit more severe symptoms than females. This conclusion is compatible with an Iran report in 2019 (*n* = 201) [22].

This observation is consistent with a 20-year research study conducted in Spain, which reported that up to 85.9% of fatalities resulting from Hymenoptera stings occurred in males [23]. Another study in a rural Mediterranean population from Eastern Spain also noted that males predominantly experienced large local reactions [24]. A study in Sweden revealed a positive correlation between sensitization to bee or wasp venom and male gender [25]. Importantly, this association is not limited to Europe; investigations in the United States, Canada, and Australia have also identified male sex as a risk factor for severe anaphylactic reactions due to Hymenoptera stings [26].

### 4.4. Gender Differences in Hymenoptera Sting Number and Severity of SRs

As noted in previous studies conducted in Taiwan and Italy, the number of stings has been identified as a significant risk factor influencing the outcome of anaphylactic reactions caused by Hymenoptera stings [16, 27]. A study from Thailand also observed a strong association between adverse clinical outcomes—such as death, AKI requiring renal replacement therapy, respiratory failure requiring intubation, and hypotension requiring vasopressor support—and the occurrence of more than 10 bee stings [28]. Our investigation revealed a notable gender difference, with males experiencing significantly more Hymenoptera stings (1.3 vs. 1.0, *p*=0.049). This suggests a possible link between the higher number of stings in males and the occurrence of more severe SRs compared to females.

### 4.5. Gender-Based Differences in Sting Location and Risk Factors for SRs

A 2019 study from Poland found that stings were more commonly located on the H than on the T and legs [29]. Although there was no statistically significant difference between gender and sting sites (*p*=0.139), our study observed that male patients were 2.1 times more likely to experience stings on the H compared to females. Additionally, male sex, being an agricultural worker, age over 16 years, and experiencing more than five stings per year were all associated with an increased risk of developing SRs following Hymenoptera stings [24].

### 4.6. After Hymenoptera Sting, Females Drop More DBP

There have been no definitive reports in the past describing the relationship between blood pressure, Hymenoptera stings, and gender. However, lower levels of angiotensin I and II have been observed following Hymenoptera stings, leading to reduced blood pressure in these cases [30]. In our study, we found that DBP in male cases of HVP was higher than in females (76.3 vs. 70.3 mmHg, *p*=0.014). No significant differences were observed in heart rate or body temperature between genders. A 2018 study from Jordan reported that both SBP and DBP were higher in males than females, with mean differences of 18.1 mmHg and 3.6 mmHg, respectively [31]. Following HVP, the drop in SBP was more pronounced in males compared to females (130.0 vs. 133.5 mmHg), while the drop in DBP was more noticeable in females (76.3 vs. 70.3 mmHg).

Honeybee venom is composed of various peptides, amines, and enzymes, including apamin (a small neurotoxin that blocks calcium-dependent potassium channels), adrenaline, noradrenaline, histamine, serotonin, and tertiapine, all of which can provoke myocardial ischemia and significant hypotension [32]. In a 2020 study from Australia and Switzerland, Hymenoptera venom–related SRs accounted for 2.2% to 7.5% of cases [24, 33, 34].

### 4.7. Gender, Sensitization, and Risk Factors in Hymenoptera Sting Reactions

Several studies have proposed that there are no significant differences in outcomes between genders following Hymenoptera stings. A 1987 study from the Netherlands reported that in insect allergy sting challenges (SCs), 28% of cases showed a SR, while 72% had only a local reaction, with no statistical difference in age or sex [35]. Similarly, a 1997 report from Turkey found that factors such as atopy, sex, occupation, smoking, and family history of bee stings were not significantly related to the severity of SRs [33]. A 2020 study from India showed that female patients exhibited more sensitivity than males in skin prick tests, though the difference was not statistically significant [36]. In contrast, a September 2023 Australian study (*n* = 363) identified female gender, age, and the number of stings as independent risk factors for mortality in wasp sting patients [37]. However, a 1995 Swedish study (*n* = 1399) found that sensitization to bees or wasps was positively correlated with atopy (odds ratio (OR) 2.0), age (OR 2.0), and male sex (OR 1.8) [25]. The mortality rate for wasp sting patients was reported as 3.9% [37].

## 5. Limitation

Our study has some limitations. First, the body weights of most patients were not measured, making it difficult to evaluate the correlation between the severity of poisoning and body weight. Second, we did not distinguish between patients who were beekeepers and those who were tourists or visitors attacked in suburban areas. It is possible that the immune responses of beekeepers and tourists differ significantly. Third, having data over a longer period or from a wider range of regions in Taiwan could help reduce bias related to sample size.

## 6. Conclusion

Our study highlights the importance of considering gender differences in the context of HVP. While males tend to experience more stings and are at higher risk for severe SRs, females are more likely to be stung during holidays, particularly in spring and autumn. Despite these differences, there were no significant variations between genders in terms of sting locations or length of hospital stay. Based on our findings, we recommend that clinicians consider gender as a potential risk factor when evaluating and managing patients with insect venom exposures. Early identification of hypotension and systemic allergic responses in female patients may help improve outcomes.

Further research with larger sample sizes and consideration of additional variables, such as occupation and immune responses, could provide a more comprehensive understanding of these gender-based disparities and improve preventive strategies.

## Figures and Tables

**Table 1:** Gender differences in Hymenoptera venom poisoning.

	All (87, 100%)	Male (41, 47.1%)	Female (46, 52.9%)	*p* value, 2-tailed
Age (years old)	46.5 ± 17.2	44.8 ± 18.5	48.0 ± 16.0	0.282

*Season*				
Spring	(11, 12.7%)	(4, 9.8%)	(7, 15.2%)	0.011^∗^
Summer	(24, 27.6%)	(13, 31.7%)	(11, 23.9%)	0.112
Autumn	(28, 32.1%)	(11, 26.8%)	(17, 37.0%)	0.017^∗^
Winter	(24, 27.6%)	(13, 31.7%)	(11, 23.9%)	0.124
Holiday	(33, 37.9%)	(11, 26.8%)	(22, 47.8%)	0.008^∗^
Number of stings	1.1 ± 0.5	1.3 ± 0.7	1.0 ± 0.2	0.049^∗^
Head and neck	(17, 19.5%)	(11, 26.8%)	(6, 13.0%)	0.139
Limbs	(69, 79.3%)	(29, 70.7%)	(40, 87.0%)	
Trunk	(1, 1.2%)	(1, 2.5%)	(0, 0%)	

*Treatment*				
AB	1	1	0	0.001^∗^
BC	1	1	0	0.001^∗^
B	44	22	22	0.318
C	2	1	1	0.490
N	39	16	23	0.561
SBP	131.9 ± 22.0	130.0 ± 20.9	133.5 ± 23.1	0.949
MAP	92.8 ± 13.4	94.3 ± 13.8	91.4 ± 13.1	0.324
DBP	73.2 ± 11.6	76.3 ± 12.1	70.3 ± 10.4	0.014^∗^
HR	90.1 ± 16.6	91.1 ± 17.1	89.2 ± 16.2	0.543
BT (Celsius)	36.7 ± 0.44	36.7 ± 0.4	36.7 ± 0.5	0.838
Length of stay (minutes)	73.5 ± 114.4	87.9 ± 160.2	60.7 ± 42.9	0.307

*Note:* HR = heart rate (beats per minute); BT = body temperature, degrees Celsius. AB injection = adrenalin 0.3 mg and Benadryl (diphenhydramine 30 mg) injection; B injection = Benadryl (diphenhydramine 30 mg) injection; C injection = chlorpheniramine 5 mg injection; BC injection = Benadryl (diphenhydramine 30 mg) and chlorpheniramine 5 mg injection.

Abbreviations: DBP = diastolic blood pressure (mmHg), MAP = mean arterial pressure, SBP = systolic blood pressure (mmHg).

^∗^Reaches statistically significant difference.

## Data Availability

The data that support the findings of this study are available from the corresponding authors upon reasonable request.

## Nomenclature

ICD: International Classification of Diseases
ICU: Intensive care unit
ANOVA: Analysis of variance
OR: Odds ratio
